# Survival and health status of DOTS tuberculosis patients in rural Lao PDR

**DOI:** 10.1186/1471-2334-10-265

**Published:** 2010-09-10

**Authors:** Hubert Barennes, Thongdam Keophithoun, Tuan H Nguyen, Michel Strobel, Peter Odermatt

**Affiliations:** 1 Institut de la Francophonie pour la Médecine Tropicale, Vientiane, Lao PDR; 2 Swiss Tropical and Public Health Institute, Department of Public Health and Epidemiology, PO Box, 4002 Basel, Switzerland; 3 University of Basel, 4003 Basel, Switzerland

## Abstract

**Background:**

Contact tracing of tuberculosis (TB) patients is rarely performed in low-income countries. Our objective was to assess the outcome of and compliance with directly observed treatment (DOTS) of TB patients over a 3 year period in rural Lao PDR.

**Methods:**

We performed a retrospective cohort study in which we enrolled TB patients who started DOTS treatment at Attapeu Provincial Hospital. We traced, through hospital records, all patients in their residential village. We conducted a standardized questionnaire with all TB patients and performed physical and anthropometric examinations as well as evaluations of compliance through counting of treatment pills at home and at the health facilities.

**Results:**

Of 172 enrolled TB patients (sex ratio female/male: 0.52, mean age: 46.9 years ± 16.9), 26 (15.1%) died. These had a lower weight at the start (34.6 *vs*. 40.8 kg, p < 0.001) and were less compliant (91.6% *vs*. 19.2%, p < 0.001) than survivors. Low compliance was associated with poor accessibility to health care (p = 0.01) and symptomatic improvement (p = 0.02). Survivors had persistently poor health status. They were underweight (54.7%), and still had clinical symptoms (53.5%), including dyspnoea (28.8%) and haemoptysis (9.5%).

**Conclusion:**

Our study suggests a lower rate of survival than expected from official statistics. Additionally, it showed that follow-up of TB patients is feasible although the patients lived in very remote area of Laos. Follow-up should be strengthened as it can improve patient compliance, and allow contact tracing, detection of new cases and collection of accurate treatment outcome information.

## Background

Laos is a low income country with a *per capita *income of USD 500 [1]; 39% of the population is classified as poor. Mortality due to tuberculosis (TB) is estimated at 24 per 100,000 population, including HIV-co-infected patients [2]. Lao People's Democratic Republic (Lao PDR, Laos) remains a HIV/AIDS low prevalence country with an estimated 0.08% HIV sero-prevalence among the adult population [3]. In Laos, TB annual incidence and prevalence are estimated at 152 and 292 per 100,000, respectively [4]. The TB detection rate has increased from 57% in 2004 to 77% in 2006 [5].

Before 1995, tuberculosis treatment in Lao provincial hospitals was either non-existent or highly irregular [2,4]. In 1995-1996, the National Tuberculosis Program (NTP) started a stepwise introduction of directly observed treatment short-course (DOTS) with diagnosis (microscopic for acid-fast bacilli [AFB]) and treatment at the district level. Chest X-rays are not done routinely [6] and impossible in many places. Free treatment is offered to all smear-positive and to smear-negative patients suspected with severe TB disease. The TB treatment consists of an initial two months of four-drug DOTS at the provincial hospital level (hospital-based, HOSP), with financial support for subsistence during hospitalization; followed by six months of two-drug home-based (HOME) DOTS, with monthly visits to the district hospital.

No financial compensation for loss of income or transportation costs exists for TB patients at the district level. They are enrolled and registered in DOTS at district hospitals, where after initial hospital-based treatment, patients receive drugs monthly. Non-compliant patients are normally further visited by a district mobile team, however, today, these visits are rarely conducted due to a variety of reasons ranging from difficult accessibility to budget and transportation constraints.

As a result, little information is available on the TB treatment outcome in Laos. The goal of this study was to specifically assess the physical status, treatment outcome and risk factors for non-compliance of TB patients enrolled in DOTS in a chosen remote area in southern Laos. We traced and examined patients enrolled in DOTS treatment between 2002 and 2004 in the province of Attapeu.

## Methods

We performed a retrospective cohort study of TB patients in Attapeu Province South of Laos from February to June 2005.

### Study area

The study was performed in the rural province of Attapeu, located on the southern border with Vietnam and Cambodia. Attapeu province has 108,000 inhabitants (10 inhabitants per km^2^) scattered in 5 districts and 280 villages. The population is predominantly poor, illiterate and belongs to ethnic minority groups. Mean monthly household income is estimated to be 29 USD [1], and access to health care is generally limited. In the Attapeu provincial hospital, TB is the third most frequent cause for outpatient attendance (247 of 8281 in 2004) and the fifth most frequent cause of hospitalization (54 of 1632 in 2004). HIV prevalence in incident TB cases is 2% in Laos [3].

### Hospital records, patients definition and follow-up

We reviewed the records of all patients hospitalized from January 2002 to December 2004 and identified all TB patients. Tuberculosis was defined as either positive AFB (3 sputum sample examination at Attapeu Hospital and grading according to NTP criteria) or sputum negative with a suggestive chest x-ray result and a positive result of the clinical algorithm provided by the NTP [7].

From the records, we collected information on hospitalizations including symptoms, weight, treatments, length of stay, bacteriology, smear positivity and grading according to the NTP [7].

All patients were traced in their residential villages. On site, we interviewed the patient with a questionnaire which was tested on comprehensiveness and subsequently improved questions. We obtained the following information: current symptoms, chemotherapy, quality of the relationship with the health facility's medical staff, attitudes regarding treatment and compliance, and knowledge about TB. Subsequently, we performed a general physical examination.

Patients were weighed lightly clothed using an electronic SECA scale for adults (precision ± 100 g). Patients' height was measured with a 0.1 cm precision using wooden measuring boards and a measuring tape. The patient's body mass index was calculated (BMI: weight/height^2^). Adults were considered underweight if BMI was ≤ 18.5 and severely underweight if the BMI was < 16 [8]. We recorded body weight before treatment, during treatment and during home visits for patients with initially available data.

### Definition

We used definitions of the NTP for compliance and treatment achievement (completeness). A patient was defined as compliant if, at the time of the survey, the patient had taken at least 90% of the prescribed number of tablets days [7]. On the other hand, non-compliant patients (as determined by the pill counts of the NTP, cross-checked with the district hospital data) were considered defaulters. A TB treatment was regarded as completed if the patient finished at least 95% of his treatment days according to his treatment category [7].

### Clinical outcomes

Survival rate was the primary outcome. Compliance with treatment, and clinical outcome including nutritional status and symptoms were the secondary endpoints.

### Laboratory and field procedures

All patients were initially checked for AFB in their sputum by the hospital. Assessing the sputum during follow-up was done by referring the patients to the provincial hospital for the scheduled 2-5 monthly sputum checks by the NTP. NTP, with the help of Service Fraternel d'Entraide, a non-governmental organisation, was responsible for the training and regular quality assessments of the laboratory.

A parallel survey was performed at the same time to detect active TB cases and to conduct contact tracing in the patients' villages. Of the 84 villages with TB patients, 10 villages were randomly selected, and all inhabitants with a chronic cough of more than 3 weeks (and/or haemoptysis) were screened using a questionnaire approach [9]. We collected two sputum samples from patients with chronic cough, one immediately and one the following morning, according to standard procedures of the NTP [7]. Samples were collected each day and sent to the district hospital where a Ziehl-Neelsen stained slide was examined for the presence of acid-fast bacilli. In addition, a sputum smear was directly examined for the presence of *Paragonimus *eggs using a light microscope [10]. Positive patients were referred to the provincial hospital for treatment. We felt that the detection of paragonimiasis (often clinically misdiagnosed as TB) was justified as there has been some evidence of it in Laos [10].

### Data management and analysis

Data was entered in Epidata freeware (http://www.epidata.dk, Odense, Denmark) and cross-checked against original data sheets. Data analysis was carried out with Stata, version 8 (Stata Corporation, College Station, TX, USA). We used Fisher's exact test to assess associations between categorical variables, student's t-test for two normally-distributed continuous variables, log rank test to compare the durations of treatment and hospitalisation between compliant and non compliant, and between survivors and non survivors and t-test for two-sample mean-comparison of paired data for the patients weight evolution. We assessed the compliance factors. First, we analyzed the factors affecting the compliance in an univariate analysis. Second, all factors with p values ≤ 0.2 were then fitted into a multivariable logistic regression model. We considered *p *< 0.05 as significant.

All participants gave informed witnessed verbal consent. The study was approved by the Ministry of Health's Ethical Council of Medical Sciences for Health Research and the provincial health authorities. The study was performed in accordance with the Declaration of Helsinki.

## Results

A total of 172 TB patients were registered at the Attapeu Provincial hospital between 2002 and 2004. They came from 80 villages and 5 districts of the province (Samakhisay 42%, Saysetha 22%, Sanamsay 28%, Sansay 5%, Phouvong 3%). The number of patients decreased with increasing distance to Attapeu city. They started their treatment in 2002 (n = 53), 2003 (n = 51), and 2004 (n = 68).

**Figure 1:**
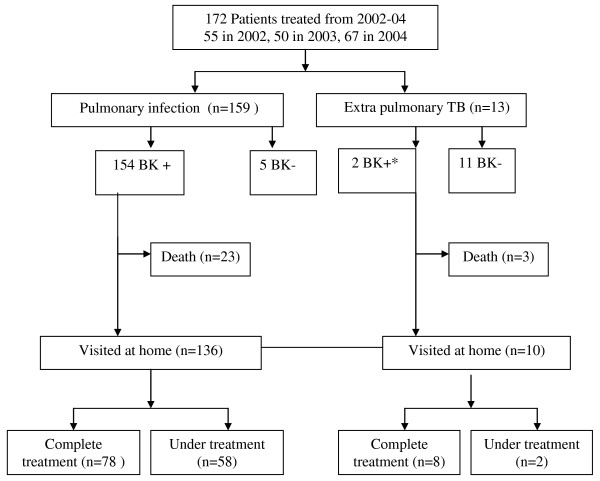
**Flow chart of tuberculosis patients through the follow up in southern Lao PDR**. * Misclassified in hospital statistics

Of the 172 TB patients, 159 patients had pulmonary TB (92.4%, Figure 1). All patients were visited at home, and the mean duration of treatment at the time of the home visit was 6 months. Table 1 and Table 2 present the main characteristics of the patients when hospitalized and during the home visits, respectively. All patients were rice farmers, living in 80 different remote villages of the province, where transportation means were scarce.

**Table 1:** Characteristics of 172 TB patients prior to treatment in Attapeu province hospital, Lao PDR

	Total	Pulmonary TB	Extra Pulmonary TB	
	n = 172	n = 159	n = 13	p
Female	59 (34.3)	52 (32.7)	7 (53.8)	0.12
Sex ratio (F/M)	0.52	0.49	1.17	0.12
Children < 15 years (%)	5 (2.9)	4 (2.5)	1 (0.7)	0.28
Age (years)	46.9 (16.9)	47.5 (1.3)	39.6 (4.2)	<0.001
Weight (kg, n = 169)	39.9 (1.4)	39.7 (0.7)	43.7 (3.1)	<0.001
BMI (n = 146)	18.2 (17.7-18.6)	18.1 (17.6-18.5)	20.1 (18.7-21.6)	0.01
Outside of the main city	93 (54.0%)	84 (52.8%)	7 (61.2%)	0.94
Median distance to Health Centre (km)	15.0 (0-87)	-	-	
Median time to Health Centre (mn)	60.0 (10-600)	-	-	
Sputum positive (n = 166)	154 (92.7%)	152/158 (96.2%)	2/8 (25.0%)	<0.001
Treatment				
2 srhz/6 he	2 (1%)	1 (0.5%)	1 (7.6%)	0.44
2 rhze/6 he	143 (83.1%)	134 (84.2%)	9 (64.2%)	<0.001
Others	27 (15.7%)	24 (15.0%)	3 (23.0%)	0.44

Mean (standard deviation) and number (rate) for continuous and discrete characteristics, respectively.

s = streptomycine, r = rifampicine, h = isoniazide, z = pyrazinamide, e = ethambuthol

**Table 2:** TB patient outcome during home visit in Attapeu, Lao PDR

	Survivors	Dead	
	n = 146	n = 26	*p*
Sex ratio (F/M)	50/96 (0.52)	9/17 (0.53)	0.97
Age (years) (95% CI)	46.2 (43.5-48.9)	51.3 (43.6-58.9)	0.1
Weight (Kg) on admission at hospital	40.8 (0.6)	34.6 (11.9)	<0.001
BMI (95% CI)	18.2 (17.7-18.6)	-	
Patients with BMI <18.5 (%)	80 (54.8)	-	
Completed treatment (%)	81 (55.4)	Not known	
Non-compliant patients (%)	28 (19.2)	22 (91.6%)	<0.001
Duration of treatment on the day of visit (days)	187.3 (3.2)	91.2 (19.3)	<0.001
Duration of hospitalization (days)	61.0 (7.8)	47.5 (3.9)	<0.001
Average missing days of treatment (days)	28.8 (4.2)	61.7(15.2)	<0.001
Physical examination			
Tiredness (%)	52 (35.6)	-	
Chronic cough (%)	68 (53.5)	-	
Haemoptysis (%)	14 (9.5)	-	
Dyspnoea (%)	38 (28.8)	-	

Mean (standard deviation) unless otherwise stated, CI confidence interval, and number and percent for continuous and discrete characteristics, respectively.

Twenty-six patients (15.1%) died (mean time to death after diagnosis: 256 days ± 304 days). Twelve patients died during hospitalization (mean time to death: 31.8 days ± 20.2 days) and 14 patients died at home (mean time to death: 81.4 days ± 69.0 days). The death rates were similar over the 3 years period (18.8%, 15.7%, 11.8%, respectively, p = 0.5). Patients who died tended to be older than the survivors, although this difference was not statistically significant (p = 0.1). Non-survivors had lower weight on admission (p < 0.001), suffered more severe symptoms, and were less compliant than survivors (p < 0.001).

At the time of our study, 80 of 146 patients (54.8%) were underweight and 30 (20.5%) were severely underweight. Patients with extrapulmonary TB had a better nutritional status than patients with pulmonary TB (p = 0.01). The evolution of the patients' weight under treatment is shown in Table 3. The body weight on hospital admission was excessively low (40 kg or less). The weight gain was more sizeable during hospitalization (+ 9.5% per month) than during home treatment (p < 0.001).

**Table 3:** Weight evolution of 72 patients under treatment with an 8 months follow-up

	Weight (kg)	95% CI	*p*
Before treatment	40.0	38.1- 41.8	
After two months of treatment	43.7	41.7- 45.7	<0.001
After 8 months of treatment	44.6^a^	42.7 - 46.5	<0.001

Mean and 95% confidence interval

^a ^Patients were selected on the basis of having both 2 and 8 month data which probably means that they are not representative of the whole cohort

**Figure 2:**
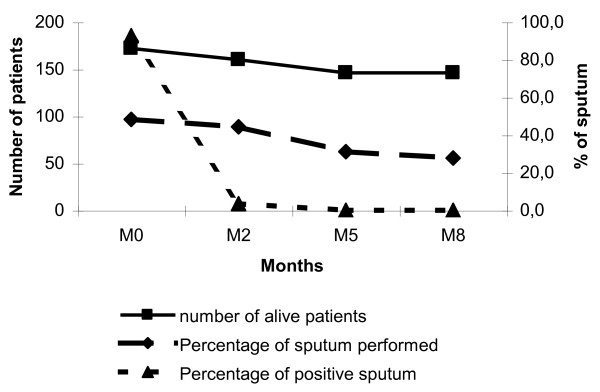
**Sputum examination during the follow-up of tuberculosis patients in Attapeu, Lao PDR**. Not all patients underwent a sputum examination which possibly underestimated the number of positive sputum over time.

We performed, at the time of DOTS enrolment, 166 (96.5%) sputum tests. At months 2, 5, and 8 after enrolment, the rates of sputum testing was 73.9% (n = 108), 63.0% (n = 92) and 55.4% (n = 81) respectively. After 2 months, 5 of 142 (3.5%) sputum follow-up examinations remained positive. Three of these were negative at 5 months, one not checked after 5 months was reported negative at 8 months of treatment, and data of one patient was not available. No sputum was reported positive after 5 and 8 months treatment (Figure 2).

Most TB patients (116 of 146, 79.5%) were heavy smokers, most of them since childhood, with strikingly high rates in males (88 of 96, 91.7%) even compared to females (28 of 50, 56.0%; p < 0.001). One-third of the patients reported their health had not improved during treatment and reported persistent symptoms as well as signs of TB during home visits (Table 2).

One patient appeared severely ill with fever, cough and general weakness when visited at home. Also, three patients had permanent dyspnoea with chest retraction, and among the 14 patients with haemoptysis, 8 had completed their treatment. We found more clinical signs in non-compliant patients (42.8% *vs*. 28.1, p = 0.04).

64 of 144 (44.4%) patients complained of difficulties with observance of the drug regime. These patients were less compliant than those who did not complain (p < 0.01). Compliant patients had a 5.2 chance of survival compared to non compliant (RR: 5.2; 95% CI: 3.2-8.5). After multivariate analysis, the main factors of non-compliance were the distance to the hospital and the feeling of general health improvement (Table 4).

**Table 4:** Reasons reported by patients for non-compliance with TB treatment, Attapeu, Lao PDR (n = 146)

	RR	95% CI	p
Long distance to health centre	8.7	1.5 - 48.5	< 0.01
Physical Improvement*	2.7	1.1- 6.6	0.02
Lack of financial resources	2.1	0.3-13.5	0.4
Lack of transportation	1.9	0.3-10.7	0.4
Need to work	1.6	0.5- 5.3	0.4
Family disorders	0.3	0.0 - 1.5	0.1

RR: relative risk, CI confidence interval

Multivariate analysis using Cox proportional hazard model

* Patients felt that they had decreased pulmonary symptoms and felt fitter

In the sub-study that was performed in 10 villages, sputum analysis from patients, close contacts and people with chronic cough were performed. Among 180 persons reporting chronic cough, we detected 5 (2.8%) adults with a TB positive sputum examination. Seven (3.9%) had *Paragonimus *sp. eggs in their sputum.

## Discussion

Our study assessed the outcome over a 3 years period of 172 registered TB patients who started DOTS treatment at the provincial hospital in Attapeu. Visits performed during the home-treatment phase allowed us to detect 28 defaulters and 26 deaths, as well as to document a large proportion of the patients (53.5%) that were still suffering from pulmonary symptoms. Nearly 10% of them were having haemoptysis, despite 8 of 14 (57.1%) having completed the treatment. Furthermore, village visits in 10 of the 80 villages allowed the diagnosis of 5 new TB and 7 new paragonimiasis cases which lived close to TB patients. Our study documents the essential role of home visits in improving a TB program aimed for remote areas.

At this time, home visits are rarely performed in practice and many efforts describing patient outcomes are only hospital-based studies [11]. Earlier studies have shown that, in remote areas, home visits can contribute substantially to achieve the WHO-recommended target of 85% cure-rate by timely identification of defaulters and close-contact tracing [8]. The effectiveness of home visits remains high even when patients are seen by non professional health-workers. Home visits also improve case detection rates by examination of close contacts. Children in close contact with patients are at particularly high risk of TB [12]. In Northern Laos, the risk of latent TB infection in children was increased 6-fold in close contacts [13]. Active case-detection among household contacts yields nine times more TB cases than passive case detection, and is an opportunity for reducing TB morbidity and mortality [14].

The proportion of sputum controls at 2, 5, 8 months was low. Health-workers who deliver the drugs monthly should also carefully check whether scheduled sputum examinations have been completed. Our follow-up study further documents the generally acknowledged advantages of home visits and quantifies the sizable amount of treatment defaulters and newly diagnosed TB patients among contacts. We argue that in low income countries such as Laos, active monitoring of DOTS TB patients - not just routine drug delivery - is particularly essential as initial efforts to diagnose and treat patients with TB would be otherwise wasted.

Our study shows that reaching patients in most remote villages is feasible even with limited resources. In Laos, this is possible because the active system of village leaders in place allows for tracing people. This feature of Laos' administration, culture and society should be used and expanded to increase TB patient follow-up locally; nevertheless, it should be reminded that in other poor rural countries, this type of social organisation may not be present.

The short time to death after diagnosis and the compromised nutritional status suggest that patients only sought care very late in the course of their disease. This reflects observations in neighbouring rural China burdened with similar patient and physician's delays. This survey gives insight into the mortality and non-compliance of patients in remote areas, which are unfortunately nearly two times higher than the national statistics [2]. Our patients were chosen out of the hospital records and might have been the more serious cases, causing in turn an overestimation of the low health status of our sample. However, in Laos all TB patients are hospitalised for the initial phase of DOTS, regardless their physical status.

Our study demonstrates the relationship between compliance during DOTS, the difficult geographical access to health services, and the patients' self perception of improvement. In rural Laos, transportation is expensive: a patient living 40 km from the health centre has a 4 USD round-trip to obtain his treatment. This is a substantial cost given a *per capita *income of 500 USD/year. Decentralization of DOTS and active follow-up at the health centre or village will increase access, and efforts to realize this improvement are currently underway. Other compliance factors which are known from other settings such as the bitterness of the drugs, the relationship and communication barriers with health-workers [15], were not of importance in our setting. Interestingly, compliance to DOTS was similar during hospitalization and home treatment periods. Hence, factors other than difficult geographical access must exist.

We conducted our study with limited resources, our results relying largely on interviews and basic physical examinations. Systematic sputum examination was performed only in a sub-sample of villages, and etiology of persistent symptoms could not be followed-up in detail; *i.e*. the role of drug resistance, treatment failure, advanced or complicated TB, associated lung injuries due to heavy smoking, or paragonimiasis, remain unknown. Paragonimiasis is endemic in Laos and is frequently overlooked [10,16]. The severity of disease at treatment onset could not be judged on records alone. Finally, smoking of locally grown tobacco is very common in rural Laos, even in children or pregnant women. Smoking during childhood is associated with a higher risk of TB, and facilitates pulmonary disease transmission [17,18]. However, in this study, we could not quantify the contribution of smoking to pulmonary disease; thus the call remains for future investigations to address this issue from the perspective of TB control, and help quantify and raise awareness about anti-smoking campaigns in rural Laos.

## Conclusion

Home visits of TB patients and contact tracing are feasible in remote rural Laos even with limited resources. The level of survival of TB patients after DOTS is probably lower than can be expected from the rate of treatment. Home visits could probably help improve physical follow-up of patients and the rate of sputum follow-up. The insight they provide into the effectiveness of the NTP is essential. Home visits are a step toward improved access to care services at the peripheral level. Further quantification of increased effectiveness of TB programs in resource poor countries through home visit and contact tracing may lead to a wider use of this practice.

## Competing interests

The authors declare that they have no competing interests.

## Authors' contributions

HB, PO, TNH, TK, MS designed the study. TK, TNH, conducted the field work. HB performed the statistical analysis and interpretation of the data together with TK, TNH and PO. HB wrote the manuscript with support from PO, TK, TNH, and MS. All authors read and approved the final version of the manuscript.

## List of abbreviations

(TB): Tuberculosis; (Laos): Lao People's Democratic Republic; (OR) odd-ratio; (95% CI): 95% confidence interval; (NTP): National Tuberculosis Program; (DOTS): directly observed treatment short-course; (HOSP): hospital-based; (HOME): home-based; (BMI): body mass index; (GDP): gross domestic product.

## Pre-publication history

The pre-publication history for this paper can be accessed here:

http://www.biomedcentral.com/1471-2334/10/265/prepub
